# Gas Chromatography–Mass Spectroscopy (GC–MS) Simultaneous Determination of Limonene, Linalool, and Linalyl Acetate in Rat Plasma Following Transdermal Administration of the Essential Oil of Bergamot Loaded Onto Solid Lipid Nanoparticles (NanoBEO)

**DOI:** 10.1002/ptr.70147

**Published:** 2025-12-08

**Authors:** Luigi A. Morrone, Laura Rombolà, Antonella Leggio, Emilia L. Belsito, Ludovica Scorzafave, Enrica De Rasis, Martina Pagliaro, Kengo Hamamura, Takafumi Hayashi, Giacinto Bagetta, Maria Tiziana Corasaniti, Damiana Scuteri

**Affiliations:** ^1^ Preclinical and Translational Pharmacology, Department of Pharmacy, Health and Nutritional Sciences University of Calabria Rende Cosenza Italy; ^2^ Department of Clinical Pharmacokinetics, Faculty of Pharmaceutical Sciences Kyushu University Fukuoka Japan; ^3^ Laboratory of Pharmaceutical Sciences, Faculty of Pharmaceutical Sciences Tohoku Medical and Pharmaceutical University Sendai Japan; ^4^ Department of Health Sciences University “Magna Graecia” of Catanzaro Catanzaro Italy

Dear Editor,

Bergamot Essential Oil (BEO, *Citrus bergamia* Risso et Poiteau) proved strong, preclinical evidence of efficacy in models of pain relevant to clinical conditions (Scuteri, Hamamura, et al. 2021), fundamentally involved in the development of agitation during dementia (Sampson et al. 2015) when misidentified (Sengstaken and King 1993), and the role of its components after administration by inhalation (Scuteri, Rombolà, Crudo, et al. 2022b) and transdermal route (Scuteri, Rombolà, Crudo, et al. 2022a) was investigated. Due to its proven effectiveness, BEO deprived of bergapten was engineered in a nanotechnology delivery system, based on solid lipid nanoparticles (SLN), and devised in an odorless cream known as NanoBEO (Scuteri, Cassano, et al. 2021) (patent n° 102019000013353; international patent deposit n° EP 4003294) for clinical study in the frame of the pilot phase of the trial BRAINAID (NCT04321889) (Scuteri, Sandrini, et al. 2021). NanoBEO confirmed the properties of BEO (Scuteri, Rombolà, Hayashi, et al. 2022), but the pharmacokinetics (PK) characterization of active components after application is necessary to deepen the knowledge concerned with the reported biological and phenotypical effects (Scuteri et al. 2024). Previous studies developed feasible and accurate gas chromatography–mass spectroscopy (GC–MS) methods for the PK determination and quantification of volatile constituents in animal plasma after EOs administration (Friedl et al. 2010; Li et al. 2018; Wang et al. 2023). Thus, the aim of the present study was to adapt a simple, fast, and reliable GC–MS method for the simultaneous determination of limonene, linalool, and linalyl acetate in plasma samples to allow the PK profile of these volatile compounds to be established after transdermal administration of NanoBEO.

## 1 Methods

Ethyl acetate, chloroform, and dichloromethane were used as solvents. Tetradecane was used as an internal standard (IS). All solvents and chemicals were of analytical grade and purchased from Sigma‐Aldrich Co. (Italy). BEO deprived of bergapten was kindly supplied by “Capua Company1880 S.r.l.”, Campo Calabro, Reggio Calabria (Italy). Particularly, BEO was used by “Officina Farmaceutica Italiana S.p.A.” and “Macrofarm S.r.l.” to produce NanoBEO, based on the methods previously described (Scuteri, Cassano, et al. 2021).

Male Wistar rats (weight 373 ± 8 g) (Charles River, Calco, Como, Italy) were maintained under conventional laboratory conditions at a temperature of 25°C ± 2°C and a 12 h natural light period. Commercial pellet diet (Charles River, Calco, Como, Italy) and tap water were provided *ad libitum*. The in vivo procedures were conducted respecting the guidelines of the Italian Ministry of Health (DL 26/2014), in conformity with the 2010/63 European Directive, and the experimental protocols (n. 676/2022‐PR) were approved by the local animal welfare organism (OPBA, University of Calabria) and by the Ministry of Health at the National Institute of Health (NIH, Rome). All animal procedures were performed in accordance with the NIH Guide for the Care and Use of Laboratory Animals and Animal Research: Reporting of in vivo Experiments (ARRIVE) guidelines 2.0 (du Percie Sert et al. 2020). On the day of the experiment, the animal, placed in a clean cage, was transferred to the laboratory for 1 h. After removing the fur, the skin of the back (2–3 cm) was cleaned with sterile saline (0.9%) and NanoBEO cream (containing 5.67 mg of BEO defurocumarinized) was administered transdermally (with a wooden spatula, and massage was performed until fully absorbed). Blood samples were collected after cream application at 0, 10, 30, 90, 180, 300, 480, 720, and 1440 min. A rat restrainer (2Biological Instrument RTV‐180) was used for serial blood sampling. In order to dilate the blood vessels, the tail was wiped with warm water and thoroughly dried. The left lateral tail vein was punctured and three blood samples were obtained from each rat. The first two blood samples were 400 μL each and were collected into heparinized syringes. The first prick was made at the tip of the tail, moving toward the base for subsequent sampling at different time points. At the time of the third and final blood sample, rats were euthanized by Iso‐Vet 2% (Piramal Critical Care SpA, Italy) inhalation, and blood was collected into heparinized syringes by cardiac puncture. Blood, collected in 1.5‐mL Eppendorf tubes containing 2% w/v dipotassium EDTA, was centrifuged at 4000 rpm for 15 min at 4°C (Heraeus Fresco21, ThermoScientific, USA) to obtain plasma. Plasma was transferred to clean tubes and stored at −80°C until it was analyzed (Kurawattimath et al. 2012).

## 2 GC–MS Analysis

A stock solution containing 10 μL of each analyte (limonene, linalool, and linalyl acetate) was prepared to allow for quantitative analysis. It was made up to a volume of 5 mL with dichloromethane. The final concentration of the analytes in the stock solution was as follows: limonene 1.67 mg/mL; linalool 1.64 mg/mL; linalyl acetate 1.74 mg/mL. From this solution, two solutions A and B were prepared by means of subsequent dilution. Solution A was obtained by taking 0.1 mL from the stock solution, making it up to a volume of 2 mL with dichloromethane; the final concentrations of the analytes were as follows: limonene 0.083 mg/mL; linalool 0.082 mg/mL; linalyl acetate 0.087 mg/mL. Solution B was obtained by taking 0.1 mL from solution A, making it up to a volume of 2 mL with dichloromethane; the final concentrations of the analytes were as follows: limonene 4.175 × 10^−3^ mg/mL; linalool 4.1 × 10^−3^ mg/mL; linalyl acetate 4.35 × 10^−3^ mg/mL. Three stock solutions were then prepared by taking 10, 30, and 60 μL respectively from solution B, making it up to a volume of 10 mL with dichloromethane. The final concentrations of the three stock solutions are reported:

Stock 1: limonene 4175 ng/mL; linalool 4.1 ng/mL; linalyl acetate 4.35 ng/mL;

Stock 2: limonene 12.525 ng/mL; linalool 12.3 ng/mL; linalyl acetate 13.05 ng/mL;

Stock 3: limonene 25.05 ng/mL; linalool 24.6 ng/mL; linalyl acetate 26.1 ng/mL.

The IS was added to each stock solution. Calibration curve standards were prepared by spiking plasma samples from untreated animals with standard solution, with at least 6 concentration levels of calibration standards. The analyses of the different plasma samples were carried out in triplicate. Quantitative data were obtained by comparing the analyte/IS area ratios in the standard solutions with the corresponding ratios in the plasma samples solutions.

Ten microliter of IS solution was added to each plasma sample (100 μL) followed by protein precipitation with 100 μL of acetonitrile; after centrifugation for 10 min at 1000 rpm at 4°C (centrifuge 5430 R, Eppendorf, Italy), the supernatant was retained as “acetonitrile extract” for GC–MS analysis while the precipitate was further extracted with 100 μL of dichloromethane, centrifuged at 1000 rpm at 4°C for 10 min and the resulting supernatant collected as “dichloromethane extract” for GC–MS analysis.

The GC–MS analyses were conducted using an Agilent Technologies GC–MS instrument equipped with HP6890 gas chromatograph and HP5973 mass spectrometer, using HP‐5MS capillary column (dimethylpolysiloxane containing 5% phenyl substituents, 0.25 mm internal diameter, thickness film 0.25 m, length 30 m), operated in electron impact ionization mode (−70 eV). Helium was used as carrier gas with a flow rate of 1 mL/min. The GC–MS analyses were carried out in splitless mode (splitless time 1 min). The following column temperature program was used in all GC–MS analyses: 50°C for 1 min; increase of 25°C/min up to a temperature of 280°C, maintained isothermal for 10 min.

## 3 Results

To perform the simultaneous extraction of limonene, linalool and linalyl acetate, the extraction capacity of different solvents was evaluated, including ethyl acetate, chloroform and dichloromethane. From the analyses conducted the extraction in dichloromethane had the best yield, so it was chosen as the extraction solvent to conduct the analyses. Before extraction with organic solvent, the samples were treated with acetonitrile to favor the precipitation of plasma proteins that could influence the results. The quantitative GC–MS analysis was carried out in Selected Ion Monitoring (SIM) mode, monitoring specific ions for each analyte. The identification of the compounds was based on comparison of their retention times with those of authentic samples.

**FIGURE 1:**
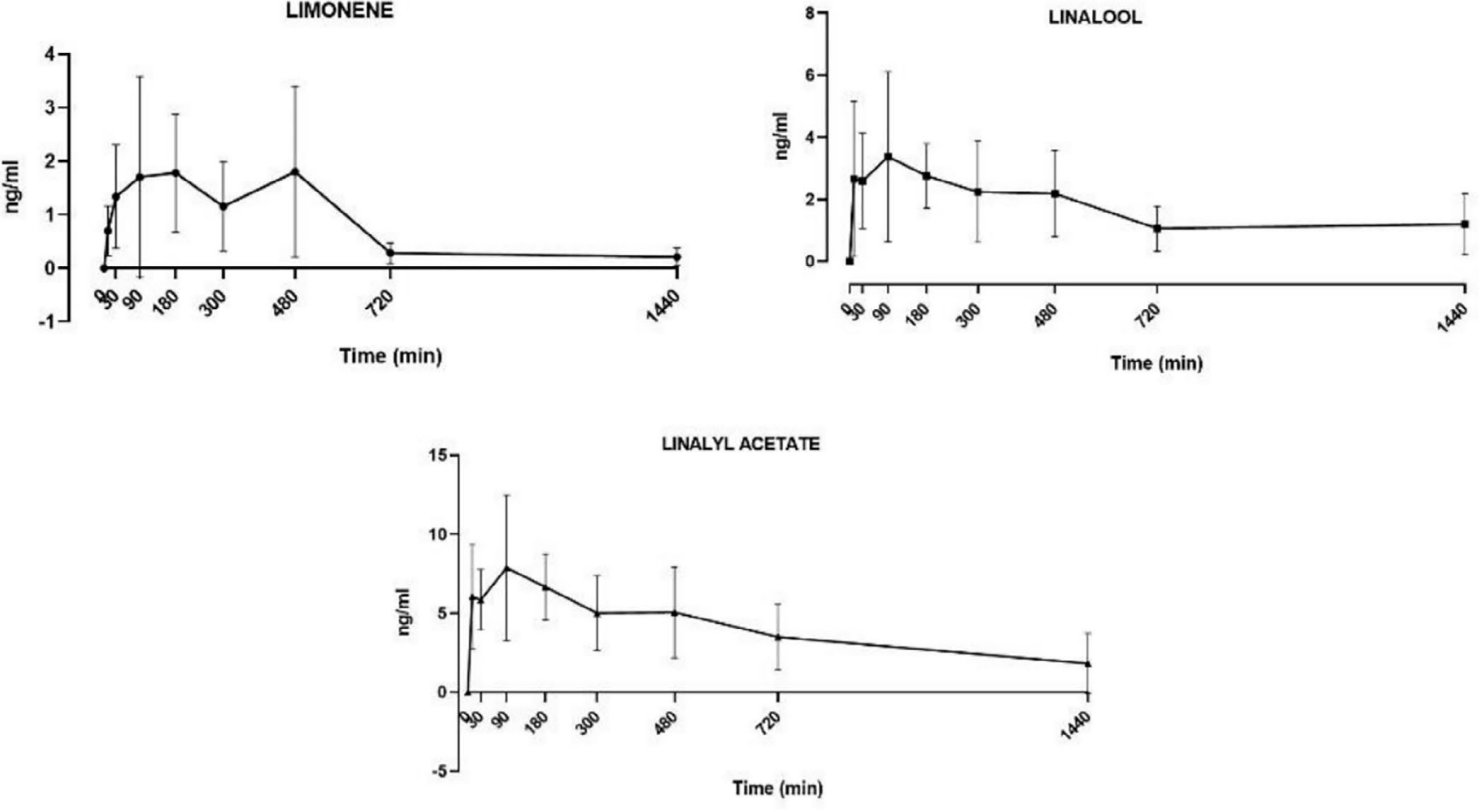
Mean plasma concentration‐time profiles of limonene, linalool, and linalyl acetate (*n* = 3–15) after dermal administration of NanoBEO in rats. Pharmacokinetic (PK) data are expressed as mean ± SD using Graph‐Pad Prism software (version 9.0, GraphPad Software, San Diego, CA, USA).

The GC–MS method described above was applied to the PK study of the three components under scrutiny after transdermal application of the NanoBEO cream. The mean plasma concentration‐time profile of limonene, linalool, and linalyl acetate is shown in Figure 1 and the main PK parameters are summarized in Table 1. For limonene, the graphical approach suggests a *T*_max_ reached between 90 and 180 min, whilst the calculated value was obtained after 480 min. This is at variance with the other two components, for which a homogeneous 90 min value was measured. The highest maximum concentration (*C*_max_; ng/mL) is measured for linalyl acetate (7.89 ± 4.6) as compared to linalool (3.43 ± 2.74) or limonene (1.80 ± 1.6) (Figure 1); AUC and Vd are shown in Table 1.

**TABLE 1:** Pharmacokinetic (PK) parameters of limonene, linalool, and linalyl acetate after transdermal administration of NanoBEO in rats.

	*T*_max_ (min)	*C*_max_ (ng/mL)	AUC 0 − ∞ min*ng/mL	Vd L/kg	Cl (mL/min/kg)	Half‐life (min)
Limonene	480	1.80	1244.23	3812	6567.15	402.43
Linalool	90	3.43	3415.92	294	232.33	845.27
Linalyl acetate	90	7.89	7507.66	684	703.14	672.97

*Note:* PK parameters were derived from 3 to 15 independent experiments with the validated software Phoenix Win‐Nonlin 8.6 (Certara, L.P., Princeton, New Jersey, USA). To estimate the best fit for the terminal phase rate constant Lambda z (λz), Phoenix repeats regressions of the natural logarithm of the concentration values using at least three data points with non‐zero concentrations. For linalool and linalyl acetate, the software used 5 and 3 points in calculation, respectively. For limonene, the mean plasma concentration is used (time range 300–1500 min), and Phoenix employed 4 points in the calculation of λz. The following PK parameters were calculated by Phoenix WinNonlin from the plasma concentration–time data based on actual blood sampling times and on the last observed concentration: Maximum observed concentration (*C*_max_), time of occurrence of *C*_max_ (*T*_max_), area under the concentration–time curve from time 0 to infinity (AUC 0 − ∞), half‐life, volume of distribution (Vd), and clearance (Cl). AUC 0 − ∞ was calculated by Phoenix using the linear and logarithmic trapezoidal method. To calculate half‐life, Phoenix used the formula “ln(2)/λz”. The estimate of the volume of distribution by Phoenix was based on the last observed concentration by the formula “MRTINF × Cl” where the parameter “MRTINF” is the mean residence time extrapolated to infinity and “Cl,” the total body clearance for extravascular administration, calculated by the formula “dose/AUC 0 − ∞.”

## 4 Conclusions

The present PK data demonstrate that limonene peaks more slowly (4 h after the administration) than linalool and linalyl acetate (peaking after one hour and a half). This and other apparent differences described below cannot be explained on the basis of minimal ponderal differences of the individual components (i.e., 0.25 mg for linalool, 1.6 mg for linalyl acetate and 2.5 mg for limonene) present in a dose of NanoBEO cream. Linalool and linalyl acetate contain polar functional groups (alcohol and ester, respectively), increasing their water affinity, and these structural and physical characteristics may allow a more rapid dissociation than limonene from the SLN. In fact, at variance with the former two, limonene is a volatile, nonpolar hydrocarbon, resulting in the most lipophilic, and this may conceivably delay the peak concentration to be reached. This latter concept may also be reflected in the lowest *C*_max_ (ng/mL) measured for limonene (1.80 ± 1.6) as compared to linalyl acetate (7.89 ± 4.6) and linalool (3.43 ± 2.74) despite their starting ponderal differences (see above). Therefore, limonene was absorbed less efficiently and in a slower manner than linalool and linalyl acetate after transdermal administration of NanoBEO, supporting the deduction that SLN form an efficient reservoir in the dermal structures from which the components under scrutiny dissociate differentially. The structural characteristics of the SLN seem to condition also the half‐life of the three components ranging from 6,5 (limonene) to 14 (linalool) hours. However, the former seems to find justification in a more efficient clearance for limonene as compared to linalool and linalyl acetate (see Table 1). Appreciable differences are also observed in the extent of the Vd, that of limonene being 6–12 fold greater than that of linalool and linalyl acetate, respectively. Whether the latter parameter needs to be corrected for confounding factors (the sequestration of limonene within the SLN or dermal structures) needs to be further investigated. However, it is very important to note that the formulation of NanoBEO yields very low plasma concentrations of each individual component, and these do not seem to limit the efficacy of NanoBEO reported in clinic (Scuteri et al. 2024). This is of the utmost importance in the light of available data concerned with Silexan, a proprietary essential oil manufactured by steam distillation from *Lavandula angustifolia* flowers, whose PK is well characterized and approved for application in behavioral disturbances (Müller et al. 2021). Even in the light of objective difficulties generated by differences in formulation (SLN vs. hydrolate), dosage (5.67 mg [0.01 mg/kg] vs. 80 [1 mg/kg] or 160 mg), and route (dermal vs. oral) of administration of the two preparations, some useful considerations are possible. In fact, in a Phase I study in healthy volunteers, peak plasma levels of linalool after single oral doses of 80 mg or 160 mg Silexan (the doses used in the clinical studies) were 27 and 97 ng/mL, respectively, reported steady following repeated administration. These doses are 13 and 26 fold higher than those of BEO contained in the NanoBEO cream and, consistently, yield some 8–30 fold higher *C*_max_ for linalool, respectively. Differences in the starting dose and route of administration may conceivably explain the observed differences in the *C*_max_ values. However, the most interesting observation is that clinical safety and efficacy on agitation and pain of NanoBEO in severe dementia (Scuteri et al. 2024) occurs at a very low *C*_max_ of the main ingredients, and this makes NanoBEO a unique health technology that deserves further research and development.

## Author Contributions

**Luigi A. Morrone:** conceptualization, methodology, data curation, formal analysis. **Laura Rombolà:** methodology, data curation, formal analysis. **Antonella Leggio:** data curation. **Emilia L. Belsito:** data curation. **Ludovica Scorzafave:** data curation. **Enrica De Rasis:** data curation, formal analysis. **Martina Pagliaro:** data curation, formal analysis. **Kengo Hamamura:** data curation. **Takafumi Hayashi:** data curation. **Giacinto Bagetta:** conceptualization, data curation, writing – review and editing. **Maria Tiziana Corasaniti:** conceptualization, data curation, writing – review and editing. **Damiana Scuteri:** conceptualization, methodology, data curation, writing – original draft, writing – review and editing.

## Funding

This research is coordinated by D.S. and received partial financial support from: (1) Phase 2 RIABEO Funding (Executive Decree n.6790 of 22 June 2022) Progetto Ingegno POR Calabria FESR 2014/2020—Azione 1 1 5—Sostegno all'Avanzamento tecnologico delle Imprese Attraverso il Finanziamento di Linee Pilota e Azioni di Validazione Precoce di Prodotti e di Dimostrazione su Larga Scala (DDG N. 12814 DEL 17 October 2019); (2) the Italian Ministry of Health: NET‐2016‐02361805 (WP 5); (3) PRIN 2022 PNRR (Project code P2022 CJNW). We acknowledge funding of a PhD bursary to M.P. from Next Generation EU, in the context of the National Recovery and Resilience Plan, Investment PE8—Project Age‐It: “Ageing Well in an Ageing Society” co‐financed by Next Generation EU [DM 1557 11.10.2022]; SICURA‐DSLN funding (Executive Decree a DDG N.8895 del 19/06/2025 e DDG N.9138 del 24/06/2025) Avviso pubblico Ricerca e Sviluppo Funding ‐ PR CALABRIA FESR FSE 2021‐2027 ‐ PRIORITA' 1 ‐ Una Calabria più competitiva e intelligente ‐Avviso pubblico “Azione 1.1.1 Sostegno a progetti di attività di ricerca, sviluppo e innovazione, anche in collaborazione con organismi di ricerca, nelle Aree e nelle traiettorie prioritarie della S3”.

## Ethics Statement

The authors have nothing to report.

## Consent

The authors have nothing to report.

## Conflicts of Interest

The authors declare no conflicts of interest.

## Data Availability

The original contributions presented in this study are included in the article; further inquiries can be directed to the corresponding author.
